# Cessation of exclusive breastfeeding and predictors among infants aged 0–6 months in Ararso district of the Somali region, Ethiopia. A community-based cross-sectional study

**DOI:** 10.7717/peerj.15963

**Published:** 2023-09-25

**Authors:** Kalid Hassen Ibrahim, Abdulkarim Mohammed Ali, Zelalem Tafese Wondimagegne

**Affiliations:** 1 College of Dry Land Agriculture Food Science and Nutrition Program, Jigjiga University, Jigjiga, Somale Regional State, Ethiopia; 2 School of Nutrition Food Science and Technology, Hawassa University, Hawassa, Sidama, Ethiopia

**Keywords:** Exclusive breast feeding, Ararso district, Ethiopia, Cessation, Knowledge

## Abstract

**Background:**

Exclusive breastfeeding (EBF) is the gold standard of child feeding practice in which the infant only receives breast milk without any additional food or drink, not even water and it lasts up to 6 months after delivery. In the study area, there is a lack of data on the prevalence of cessation of EBF.

**Methods:**

Community-based cross-sectional study design was used among 292 mothers of infants aged 0–6 months. The data was collected using a pretested structured questionnaire. Bivariate and multivariate logistic regression analyses were carried out. An odds ratio with a 95% confidence interval was used to measure the strength of the association. Statistical significance was declared at *P*-value <0.05.

**Results:**

The prevalence of cessation of EBF was 57.3% with 95% CI [50.9–62.6]. This study showed being employed (working outside the home) (AOR = 2.44; 95% CI [1.32–4.53]), being rural residence (AOR = 1.87; 95% CI [1.05–3.32]), and inadequate knowledge of EBF (AOR = 2:02; 95% CI [1.19–3.43]) were independent predictors of cessation of EBF.

**Conclusion and Recommendation:**

Our data identified a higher prevalence of cessation of EBF in the study area compared to most studies in Ethiopia and elsewhere. Efforts on improving knowledge of the importance of EBF particularly in rural areas and support for breastfeeding-employed women are recommended.

## Introduction

Exclusive breastfeeding (EBF) is a practice in which the mother gives her baby only breast milk and nothing else (Ahmed et al., 2020; UNICEF, 2018a). Globally about 44% of infants are exclusively breastfed during their first four months of life (Fetene et al., 2016; WHO/UNICEF, 2021). In sub-Saharan Africa, there is a regional variation in the prevalence of EBF ranging from 23.7% in Central Africa to 56.6% in Southern Africa (UNICEF/WHO, 2017). A recent review article revealed in Ethiopia the pooled prevalence of EBF at 6 months was 60.42% (Wake & Mittiku, 2021).

As recommended by WHO and UNICEF all mothers should exclusively breastfeed their babies for the first six months of life and then complement them with suitable, safe, and proper complimentary food after 6 months of age (UNICEF, 2018b). Ethiopia reported that only 59% percent of infants less than six months of age are exclusively Breastfed (EPHI/ICF, 2019). The government emphasizes the promotion of EBF as one goal of health extension programs to enhance proper infant and young child feeding in Ethiopia (FMOH, 2021). Nutrition program implementers in Ethiopia are working to improve optimal breastfeeding in the country (WHO, 2012). Among them, WHO and UNICEF are the two most supportive global breastfeeding projects targeting to increase EBF by at least 50% by 2025 (Zong et al., 2021).

Besides the infant, the mother can also be benefited from immediate breastfeeding in reducing the risk of postpartum hemorrhage and an advantage in lactational amenorrhea for family planning in the first six months after delivery (Dukuzumuremyi et al., 2020),

Breastfeeding initiation prevalence is always higher in the first hour of delivery and decrement in the EBF is reported after a while. A study conducted in Addis Ababa and Northwest Ethiopia revealed that 58.3% and 77.7% of mothers initiated breastfeeding within one hour of birth (Ekubay, Berhe & Yisma, 2018; Ayalew et al., 2022).

Prior research findings show that the introduction of solid foods and drinks before 6 months of age is common practice in poor as well as middle-income countries (Kavle et al., 2017; Gatica et al., 2021). Increasing the rate of breastfeeding would save the lives of more than 820,000 children under 5 years of age (WHO/UNICEF, 2021). Infant respiratory and gastrointestinal-related morbidities can also be reduced by promoting the practice of EBF during the first 6 months of life (Lee & Binns, 2020). When infants are not exclusively breastfed for the recommended six months, they may have a high likely hood of diseases including diarrhea, gastrointestinal tract infection, allergic diseases, diabetes, and obesity (Martin, Ling & Blackburn, 2016). Diarrheal-related mortalities are high and common in sub-Saharan countries and children in this region can be prevented by initiation and longer duration of exclusive breastfeeding (Ogbo et al., 2017). Cessation of EBF increases the risk of pneumonia and diarrhea morbidity (Nigatu, Azage & Motbainor, 2019), and growth falters when compared to those maintained EBF (Kaushal et al., 2017). Additionally, breastfeeding benefits society by reducing health care costs, parental employee absenteeism in taking health care of the child, and associated loss of family income (Tsegaye et al., 2019). EBF has numerous health benefits for mothers including lowering the risk of breast and ovarian cancer, type 2 diabetes, postpartum depression, and having a long duration of amenorrhea or as a natural family planning approach (Dukuzumuremyi et al., 2020; Unar-Munguía et al., 2017). In general, expanding the practices of EBF is a critical component of achieving the 2030 sustainable development goal (Ahmed et al., 2020).

Cessation of exclusive breastfeeding can result from a variety of reasons including maternal socioeconomic features, psychosocial factors, and exclusive breastfeeding experiences (Tsegaye et al., 2019). Since these factors may be different from region to region it is critical to comprehend all factors related to the cessation of EBF practices to educate, promote and advance the effectiveness of adequate EBF practices among mothers.

A variety of factors were reported by previous studies in Ethiopia and elsewhere for cessation of EBF including maternal age over 35 years (Brown et al., 2013) and younger women with 19 years and less (Ogbo et al., 2017; Yeneabat, Belachew & Haile, 2014; Teka, Assefa & Haileslassie, 2015). Other studies reported maternal employment (Alina, Manan & Isa, 2013; Mekuria & Edris, 2015; Ratnayake & Rowel, 2018); lower educational status of the mother (Ratnayake & Rowel, 2018; Khanal et al., 2014; Asfaw, Argaw & Kefene, 2015; Tang et al., 2019); urban dwelling (Woldie & Edris, 2014; Fombong et al., 2016); inadequate knowledge on EBF (Khanal et al., 2014; Fombong et al., 2016; Bayissa et al., 2015); place of delivery (Fombong et al., 2016; Tadesse, Mesfin & Mekonnen, 2016; Nukpezah, Nuvor & Ninnoni, 2018); caesarean delivery (Al Ghwass & Ahmed, 2011; Sodeno et al., 2021); lack of post-natal care (Alina, Manan & Isa, 2013; Mekuria & Edris, 2015); lack of breast feeding counseling (Mekuria & Edris, 2015; Ratnayake & Rowel, 2018; Idris & Elgorashi, 2015; Aidam, Pérez-Escamilla & Lartey, 2005); initiation of breast feeding (Inano et al., 2021; Amanuel, Misgan & Amit, 2019); being pregnant for the first time (Cato et al., 2017; Kelkay et al., 2020); and the role of marriage partners, grandmothers, and health care providers (Kelkay et al., 2020; Chang et al., 2019; Adugna et al., 2017; Van Dellen, Wisse & Mobach, 2019; Leah, Willy & Patrick, 2017; Brand, Kothari & Stark, 2011) as important factors associated with cessation of EBF.

In the study area, there is inadequate data on the prevalence of cessation of EBF and associated factors. As EBF is the strongest predictor of baby survival it is very crucial to determine the prevalence and predictors to formulate contextual cost-effective interventions. Therefore this study intended to determine the prevalence of cessation of EBF and identify important factors that play a role in mothers’ cessation of EBF.

## Methods and Material

### Population

The source population was all permanent inhabitant mothers and children under 6 months of age months children working in both governmental and non-governmental organizations in the Arrarso district. The study population was randomly selected mother-child pairs from the permanent inhabitant mothers in the Ararso district under 6 months old during the study period and willing to participate in the study.

## Study design and area

**Figure 1:**
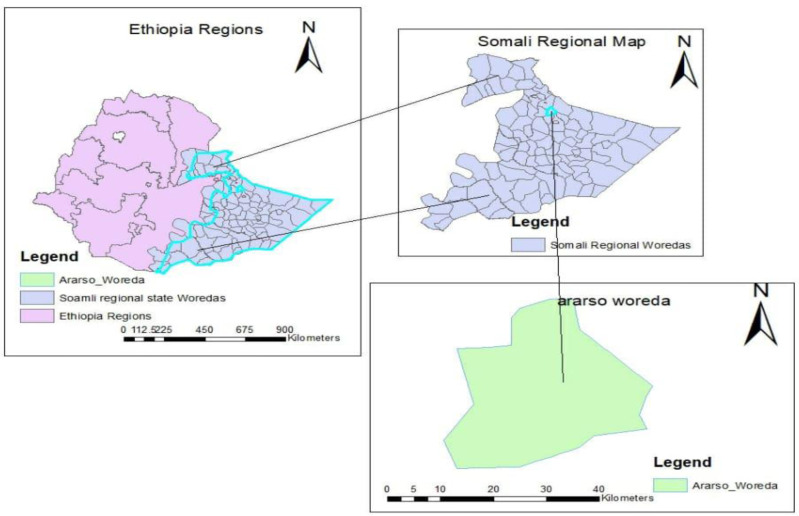
A map showing the study area.

A community-based cross-sectional study design was carried out to assess exclusive breastfeeding cessation and associated factors among mothers of infants aged 0–6 months in Araarso district, from September 2019 to June 2020. According to the report of the district administration office (Arrarso District Annual Report, 2020), the total population size is 89,174 both in urban and rural areas out of which 46,685 are male, 42,489 females and almost all (99.7%) are Muslim religion followers (Fig. 1).

### Sample size determination

The sample size was determined by using the single population proportion formula considering a 95% confidence level, a margin of error of 5%, a maximum proportion (p) of 50%, and the initial sample size was 384. After correction for finite population, since the total number of infants whose age is between 0-6 months living in the selected district is 5,380 and including 5% for a non-response, the final sample size was 292 infants aged 0–6 months with their mothers.

### Data collection

The district has ten kebeles (the smallest administrative unit in Ethiopia); four kebeles were selected, two kebeles from the town and two from the rural area by using a lottery method. The sample size was proportionally allocated to the selected Kebeles based on the number of children 0–6 months in each kebele. Study participants were selected by using a systematic random sampling technique with a sampling interval (K) of three. In the absence of an eligible respondent in a given household, substitution has been made by an individual in the next household.

### Data collection tool and technique

Data were collected using a pre-tested, structured, and interviewer-administered questionnaire which was adapted from previous studies (CSA/ICF, 2016; Adugna et al., 2017; Asfaw, Argaw & Kefene, 2015; Nigatu, Azage & Motbainor, 2019). The data collection tool includes three sections; the first section is about sociodemographic characteristics. These questionnaires were used to collect information from all study participant mothers on sociodemographic, economic, and related characteristics. The second and third parts of the questionnaire were detailed analyses related to the knowledge and attitude related to the cessation of EBF that followed the scalar scoring method (Sharma & Chalise, 2018). There was a separate set of questionnaires having four questions for measuring the knowledge of the mothers on EBF. Each question carried a score of one for a correct/favorable answer and zero for an incorrect/unfavorable response. Finally, the total score for each group was added separately and ranked. A rank of ≥60 percent of responses was considered adequate knowledge or a favorable attitude. This grading was designed by the researchers as there was no standard scoring in the literature. The content, reliability, and validity of the questionnaire were then assessed by knowledgeable and well-experienced lecturers and nutrition specialists at Jigjiga University for acceptable accuracy. The questionnaire was pre-tested on 5% of the sample size among healthcare workers at the health center which is found 10 km away from the study area and some corrections were made accordingly. Postnatal care service utilization was the outcome variable for this study. It is a binary outcome variable. Study participants were asked, “Whether they utilized postnatal care or not at least once in the most recent birth”. They answered either “Yes” or “No”. Similarly, the mother was asked whether she got breastfeeding counseling at least once in the most recent pregnancy or after birth. Pre-testing was done in a similar group of population in another area with a similar sociodemographic situation.

### Data collection procedures

Data were collected through face-to-face interviews with the mother at home. The questionnaire was pre-tested in five percent of the study subjects before the actual data collection in selected kebeles to ensure the validity, reliability, and other related data collection procedures standard.

### Ethics

The ethical approval for this study was obtained from the Ethical Review Board of the School of Graduate Studies, Jigjiga University GSR/0423/12. Informed verbal consent was also obtained from the respondents, after the necessary explanation about the purpose, benefits, and risks of the study and also their right on the decision of participating in the study.

### Quality control

Data collectors and supervisors were trained for two days on the study objectives, tools, and ethical procedures. The supervisor and primary investigator closely followed up on the data collection activities, ensuring completeness and ethics in data collection. Data confidentiality was confirmed by omitting the name of the study participants from the questionnaire and reassuring the safety of the place where the questionnaire will be stored after data collection.

### Statistical analysis

Data were cleaned and entered into a database using Epi Data Version 3.1 and then exported to SPSS to Statistical Package for the Social Science (SPSS) version 21 (IBM Corporation, Armonk, NY, USA) for analysis. Exclusive breastfeeding cessation was dichotomized as a category into yes and no. Descriptive analysis was done to determine means, frequencies, and percentage distributions for the variables. A bivariate analysis was applied to see the crude effect of each independent variable for the exclusive breastfeeding cessation group. The normality of the data distribution was checked with the Kolmogorov–Smirnov test (Mishra et al., 2019). The model fitness was also checked by the Hosmer-Lemshow goodness of fit test, and variables with *P*-values of 0.2 were selected for multivariable logistic regression analysis. A stepwise backward elimination procedure was used in the multiple logistic regressions. The association between the dependent and the independent variables was measured using an odds ratio (OR) with a 95% Confidence Interval (CI) and a *P*-value of <0.5 used to report statistical significance,

### Operational definition

**Exclusive breastfeeding**: Feeding only breast milk except for medications, expressed breast milk, ORS, and vitamins when indicated.

**Exclusive breastfeeding cessation**: A mother who stops or interrupts EBF her child before 6 months of age, and gives the child other food or fluid with breast milk.

**Knowledge of mothers on EBF:** The mother’s responses on knowledge variables were computed and who scored less than sixty percent were labeled as inadequate and those who scored above as having adequate knowledge.

**The attitude of mothers towards EBF:** The mother’s responses on attitude variables were computed and who scored less than sixty percent were labeled as unfavorable and those who scored above as having favorable attitude.

## Results

### Socio-demographic characteristics

Among the total 292 study participants included in the study 281 (96.2%) response rate was obtained. One hundred fifty-three (54.4%) of the participants were male and children under one month’s age were 102 (36.3%). One hundred forty-four (51.2%) of the mothers were within the age range of 15–25 years (Table 1). The result also revealed almost all respondents were married (271; 96.4%) and Muslims (280; 99.6%).

**Table 1:** Demographic and socio-Economic characteristics of mothers of children 0 to 6 months old, Araarso District, of Somali Regional State, Ethiopia, 2021 (*n* = 281).

**Variable**	***n***	**Percent (%)**
**Sex of the child**		
Male	153	54.4
Female	128	45.6
**Age of the child**		
Up to 1 month	102	36.3
2–4 month	92	32.7
5–6 month	87	31.0
**Mothers of children who stopped EBF**	161	57.3
**Age of the mother during interview**		
15–25	144	51.2
26–35	123	43.8
>36	14	5.0
**Marital status**		
Married	271	96.4
Divorced	6	2.1
Widowed	4	1.4
**Educational status of the mother**		
No formal education	168	59.8
Primary school	79	28.1
Secondary school and above	34	12.1
**Occupational status of the mother**		
House wife	186	66.2
Daily laborer	62	22.1
Farmer	1	.4
Government organization employee	28	10.0
Private organization employee	4	1.4
**Monthly income of the household in ETB**		
1500–2500	94	33.5
2500–3500	82	29.2
>3500	105	37.4
**Residence**		
Rural	136	48.4
Urban	145	51.6
**Ethnicity**		
Somali	271	96.4
Oromo	3	1.1
Amhara	5	1.8
Others	2	.7
**Religion**		
Muslim	280	99.6
Christian	1	.4

**Notes.**

1 USD=41 ETB

### Obstetric and health-related characteristics

Half of the mothers 135 (48%) gave birth at the health facility, and most of them 200 (71.2%) had a vaginal delivery. More than half of the respondents 153 (54.4%) had postnatal care visits at least once during their most recent pregnancy, and half of the respondents 134 (47.7%) received counseling on infant and young child feeding (Table 2).

**Table 2:** Exclusive breastfeeding and health related factors among mothers of children 0 to 6 months old, Araarso District, of Somali Regional State, Ethiopia, 2021 (*n* = 281).

**Variable**	***n***	**Percent %**
**Place of delivery**		
Home with families	110	39.1
Health facility	135	48.0
Home wit traditional birth attendants	36	12.8
**Mode of delivery**		
Spontaneous vaginal delivery	200	71.2
Cesarean section	81	28.8
**Number of birth**		
1	188	66.9
2–4	54	19.2
≥5	39	13.9
**PNC**		
No	128	45.6
Yes	153	54.4
**Breastfeeding Counseling**		
No	132	47.0
Yes	149	53.0
**Mothers who initiate breastfeeding early**	146	52.0
**Practice EBF during interview**	120	42.7
**EBF ceased at time of the interview**	161	57.3
**Time to cessation of EBF**		
Within the first month	61	21.7
2–4 months	129	46.0
5–6 month	91	32.3
**Reason for early cessation of EBF**		
Advised by relatives/friends/neighbors	2	0.6
Going back to work	36	11.8
Mothers illness	33	13.0
Infant illness	82	29.2
Decreased breast milk secretion	128	45.3

**Notes.**

Parity: number of previous pregnancies of >20 weeks.

PNC Post natal care TBA’s traditional birth attendants EBF exclusive breast feeding

### Cessation of EBF and related factors

Our data showed that 120 (42.7%) mothers practiced EBF at the time of the interview (Table 2). EBF was ceased at the time of the interview in 161 (57.3%) of the mothers. Out of this 112 (69.5%) were breastfeeding their child with another breast milk substitute and 49 (30.5%) of the respondents had ceased breastfeeding. Half of the mothers 74 (46%) ceased within 2–4 months of infant age, most of the feeding other than breast milk includes infant formula/powder milk (91; 56.5%), fresh animal milk (63; 39.1%), Tea (6; 3.7%), and water (1; 0.6%). Of the total of 161 mothers who had ceased EBF at the time of the interview, 128 (45.3%) mothers replied with decreased breast milk, 82 (29.2%) infant illness, 33 (13.2%) maternal illness, and 36 (11.8%) going back to work outside the home as the main reason for their cessation of EBF.

### Knowledge

A total of 150 (53.3%) of participant mothers had adequate knowledge of EBF. Only 113 (40.2%) of mothers answered the right time to initiate breastfeeding within an hour of birth while more than half of the mothers 167(59.4%) answered to be initiated within 1 to 3 h. More than half of the respondents 148 (52.7) answered discard according to colostrum feed while 133 (47.3%) gave the correct answer which is fed immediately, and only 61(21.7%) responded the right time to commence complementary feeding as 6 months of the child age, while the majority of the respondents 149 (53%) responded to start complementary food before or after 6 months age (Table 3).

**Table 3:** Knowledge of mothers of children 0 to 6 months old on exclusive breastfeeding, Araarso District of Somali Regional State, Ethiopia, 2021 (*n* = 281).

**Variable**	***n***	**Percent %**
**What is the right time to give breast milk to a child after birth**		
Immediately Within the First Hour	134	47.7
After an hour	147	52.3
**What is the right thing to do with the first milk or colostrum to anew born**		
Discard	131	46.6
Feed Immediately	150	53.4
**What is actually the right time to start complimentary foods**		
4 Months	13	4.6
5 Months	129	45.9
6 Months	139	49.5
**Foods and or fluids recommended to give a child under 6 months**		
Only Breast Milk	241	85.8
Infant Formula	1	.4
Any kind of food	39	13.9

### Attitude

A total of 161 (57.3%) mothers showed a favorable attitude towards EBF. The majority of 209 (74.4%) of mothers agreed that initiating breastfeeding immediately after birth is important and 25 (8.9%) of mothers strongly agreed while only 47 (16.7%) disagreed. One hundred one (35.9%) of mothers agreed discarding the colostrum is not important and only two (0.7%) of them strongly agreed, 172 (61.2%) disagreed while only six (2.1%) of the mothers strongly disagreed (Table 4).

**Table 4:** An attitude of mothers of children 0 to 6 months old towards exclusive breastfeeding, Araarso district of Somali Regional State, Ethiopia, 2021 (*n* = 281).

**Variable**	***n***	**Percent %**
**Initiate breastfeeding in hours of birth is important**		
Strongly Agree	25	8.9
Agree	209	74.4
Disagree	47	16.7
**Discarding colustrum is good practice**		
Strongly Agree	2	.7
Agree	101	35.9
Disagree	172	61.2
Strongly Disagree	6	2.1
**Commencing complementary food at six months of child age is important while continuing breastfeeding**		
Strongly Agree	2	.7
Agree	137	48.8
Disagree	140	49.8
Strongly Disagree	2	.7
**Breast milk Alone is important for the Baby in the first six months**		
Strongly Agree	2	.7
Agree	88	31.3
Disagree	184	65.5
Strongly Disagree	7	2.5

### Factors associated with cessation of EBF

The multivariate logistic regression model (Table 5) detected associations with cessation of EBF at *P* < 0.01 for maternal employment(working outside the home) and inadequate knowledge of mothers on EBF and associations at *P* < 0.05 for being rural residents. Mothers who are employed were 2.45 times more likely to cease EBF earlier than housewife mothers (AOR: 2.45, 95% CI [1.32–4.53]). Similarly, mothers who reside in rural areas were n two times more likely to cease EBF compared to those who reside in urban areas (AOR: 1.87, 95% CI [1.05–3.32]). Similarly, those mothers who had inadequate knowledge were found with a more than two times higher likelihood to cease EBF earlier than their counterparts (AOR: 2.02, 95% CI [1.19–3.43]).

**Table 5:** Factors associated with cessation of EBF among mothers of children 0 to 6 months old children, Araarso District, of Somali Regional State, Ethiopia, 2021 (*n* = 281).

**Variable**	**Early Cessation of EBF**		**COR (CI95%)**	**AOR (CI95%)**
	Yes	No		
Maternal employment				
Employed	29(30.5%)	66(69.5%)	2.18(1.292–3.677)	2.45(1.32-4.53)^**^
House wife	91(48.9%)	95(59.1%)	1	1
Residence				
Rural	47(34.6%)	89(65.4%)	1.92(1.187–3.104)	1.87(1.05-3.32)^*^
Urban	73(50.3%)	72(49.7%)	1	1
PNC				
No	45(35.2%)	83(64.8%)	1.77(1.774–2.871)	2.01 (0.99-3.87)
Yes	75(49%)	78(51%)	1	1
Ever had counseling on breastfeeding				
No	50(37.9%)	82(62.1%)	1.45(0.902–2.341)	0.82(0.44-1.55)
Yes	70(47%)	79(53%)	1	1
Early initiation of breastfeeding				
No	50(37%)	85(63%)	1.57(0.972–2.523)	1.41(0.81-2.45)
Yes	70(47.9%)	76(52.1%)	1	1
Knowledge				
Inadequate	41(31.3%)	90(68.7%)	2.42(1.498–3.983)	2.02(1.190-3.43)^**^
Adequate	79(52.7%)	71(47.3%)	1	1
Attitude				
Unfavorable	42(35%)	78(65%)	1.75(1.073–2.838)	1.32(0 .74-2.35)
Favorable	78(48.4%)	83(51.6%)	1	1

**Notes.**

1 = Reference

** Significant at *p*-value of ≤ 0.001.

* Significant at *p*-value of ≤0.05.

## Discussion

The current study revealed that the prevalence of cessation of EBF was 57.3%. This result is in line with the studies in the Alesha region in Saudi Arabia (Al-Katufi et al., 2020) and Ankesha Guagusa District, North West Ethiopia (Yeneabat, Belachew & Haile, 2014), whereby the prevalence of EBF cessation was 60% and 57.1% respectively. In contrast, it is lower than the report from studies conducted at Dukem town, central Ethiopia, (Kebede et al., 2020), Taiwan (Chang et al., 2019), and the Democratic Republic of Congo (Babakazo et al., 2015), reported more prevalence of EBF cessation 75.7%, 70.7%, and 97.2% respectively. This shows the regional variation in the prevalence of EBF cessation and these differences may appear due to socio-cultural differences in the study setting, study period, and sample size. For instance, the study in Taiwan was hospital-based and used a large sample size, and a wide range of study periods whereas other studies were mixed-method community-based studies. However, the prevalence of EBF cessation reported in the present study was higher than that had been reported in Australia (Ogbo et al., 2017), Brazil (Vieira et al., 2010), and Sri Lanka (Ratnayake & Rowel, 2018), where 38%, 39%, and 49.2% respectively. These disparities might be due to methodological differences. Some of the studies use demographic data from sub-Saharan African countries focusing on the effect of infant feeding practice on the morbidity status of children, while others focus on factors associated with discontinuation of exclusive breastfeeding in the first month of lactation and most of these studies were facility-based. Another plausible reason might be the socio-economic disparities between the study areas.

In the present study factors that have been identified to be the main predictors for cessation of EBF were maternal employment, rural residence, and inadequate knowledge of the child’s mother on EBF.

Employed mothers were more likely to cease EBF compared to housewife mothers. A similar result was reported by the finding conducted in the semi-urban sub-district of Adigrat, Tigrai, Ethiopia (Gebriel, 2020), Canada (Atchibri & Dako, 2017), Sri Lanka (Ratnayake), Bangladesh (Akter & Rahman, 2010), Taiwan (Babakazo et al., 2015), Goba district, Southeast Ethiopia (Setegn et al., 2012), and Northwest Ethiopia (Yeneabat, Belachew & Haile, 2014).

Possibly this could be because employed mothers may return to the workplace due to a short maternity leave period, and this condition may inhibit employed mothers from EBF for the recommended six months duration.

This study has also indicated a significant difference between rural and urban resident mothers concerning the cessation of EBF. Mothers of rural residents showed a higher likelihood to cease EBF as compared to mothers of urban residents. This result is in line with a study done in Bench Maji Zone, Southwest Ethiopia (Edris, Atnafu & Tafesse, 2019). This result can be attributed to many reasons, including the reason that mothers who reside in urban might have better access to health facilities whereby they get appropriate counseling on EBF and this may narrow their knowledge gap to urban inhabitants. The other reason may be a difference in belief about breast milk as a result of a knowledge gap. Rural mothers may believe that breast milk alone was not sufficient during the age of infants as the urban. This finding contradicts some other previous studies including, a study done in North Gondar Ethiopia (Dachew & Bifftu, 2014), Debre Berhan Ethiopia (Asfaw, Argaw & Kefene, 2015), and Malaysia (Tan, 2011), in which rural residents were more likely to EBF their child than urban residents. This contradicting report emphasized the need for further study to elaborate on the reason.

Knowledge among mothers is the other significant factor for the cessation of EBF. Compared to their counterpart’s higher likelihood to cease EBF was seen among mothers with inadequate knowledge of EBF. This finding was supported by a study conducted in the democratic republic of Congo (Babakazo et al., 2015), and in Ethiopia (Kelkay et al., 2020). This could be because mothers with adequate knowledge about the duration and benefits of EBF would have better EBF practice for the recommended 6 months period. Unlike in other similar prior studies, the present study did not show a significant association between EBF cessation with maternal age and other socio-demographic characteristics including marital status, religion, ethnicity, household socioeconomic position, and educational level among the study participants. This may be because the majority of the study participant in this study was homogenous concerning their socio-demographic characteristics.

This study has some limitations including cessation of EBF is better to be assessed through a longitudinal follow-up approach. But the current study was assessed through client self-reporting questions retrospectively about the entire 6 months of age on child feeding practice which was a bit challenging to recall the exact time of cessation of EBF. Furthermore, this study used a cross-sectional study design, which made it difficult to establish a causal effect relationship.

## Conclusion

The prevalence of EBF cessation before 6 mo of the child’s age is high compared to most studies in Ethiopia and elsewhere. In the present study, half of the mothers had adequate knowledge of EBF related to the right time to initiate breastfeeding after birth, and initiating foods/fluids recommended to infants. Likewise, the majority of the study participants had favorable attitudes regarding EBF. Being a rural resident, being employed, and having inadequate knowledge of EBF practices, were the independent predictors of EBF cessation in the studied community.

Therefore, it is good enough to pay adequate attention to promoting maternal knowledge on EBF through accessible means, maternity leave policies for civil servants to create a baby-friendly working environment at their place of work are emphasized. We recommend further detailed research to be carried out at the national level to evaluate the predictors of EBF cessation.

##  Supplemental Information

10.7717/peerj.15963/supp-1 Data S1 Dataset

10.7717/peerj.15963/supp-2 Supplemental Information 2 Annex Research instrument

## Additional Information and Declarations

### Field Study Permissions

The following information was supplied relating to field study approvals (*i.e.*, approving body and any reference numbers): The Jigjiga University ethical review board approved the study (GSR/0423/12).

### Data Availability

The following information was supplied regarding data availability: The raw data are available in the Supplemental File.
