# Supplementary material for: Cessation of exclusive breastfeeding and predictors among infants aged 0–6 months in Ararso district of the Somali region, Ethiopia. A community-based cross-sectional study
Source: PeerJ. 2023 Sep 25;11:e15963. doi: 10.7717/peerj.15963 (PMC10538283; doi:10.7717/peerj.15963)
Supplement: Supplemental Information 2 — Research instrument [file peerj-11-15963-s002.docx]

# Annex II The questionnaire

**SECTIONA: Child biodata**

| No | Category/question |  | Skip |
| --- | --- | --- | --- |
| 101 | Code of the respondent |  |  |
| 102 | Sex | 1.male [ ]  2. female [ ] |  |
| 103 | Date of the birth |  |  |

**SECTION B: SECTION C: MATERNAL CHARACTERISTICS, SOCIO- DEMOGRAPHIC CHARACTERISTICS**

| No | Category /question |  |  |
| --- | --- | --- | --- |
| 201 | Age of mother in completed years |  |  |
| 202 | Marital status | 1. Married [ ] 2. Divorced [ ] 3. Widow [ ] |  |
| 203 | Residence | 1. Rural  2. Urban |  |
| 204 | Have you ever attended school? | Yes  No | **If yes go to question 204** |
| 205 | If yes, what is the highest level of school you attended | 1. No formal education [ ] 2. Completed primary [ ] 3. Not completed primary [ ] 4. secondary level education [ ] 5. Certificate level training [ ] 6. Bachelor degree and Above [ ] |  |
| 206 | What is your occupation? | 1. House wife 2. Daily labor 3. Farmer 4. Student 5. Government organization employee 6. Private organization employee |  |
| 207 | **Monthly income of the household in ETB** | 1. 1500-2500 2. 2500-3500 3. >3500 |  |
| 208 | **Ethnicity** | 1. Somali 2. Oromo 3. Amhara 4. Other |  |
| 209 | **Religion** | 1. Muslim 2. Christian |  |

**SECTION C: - OBSTATETRIC AND HEALTH REALTED FACTORS**

| **No** | Category /question |  |  |
| --- | --- | --- | --- |
| 301 | Where was your child born? | 1, Home [ ]  2, Health facility []  3, At a TBA‟s house [ ] |  |
| 302 | What kind of delivery? | 1, Normal [ ]  2, Cesarean section[ ] |  |
| 303 | How many pregnancies did you have in your whole life? | …………. pregnancies |  |
| 304 | Did you receive PNC with in the first 24 hr of delivery? | Yes  No | If yes go to question number 304 |
| 305 | where did you receive, PNC care | 1. Governmental hospital 2. Private hospital 3. Health center 4. Health post 5. Private clinic |  |
| 306 | Did you receive counseling regarding, breast and complementary feeding practice during PNC service? | 1. Yes 2. No |  |
| 307 | Parity | 1. Nulliparous 2. Multiparous |  |

**SECTION D: - MATERNAL KNOWLEDGE, AND ATTITUDE TOWARDS EXCLUSIVE BREASTFEEDING**

| **No** | Category /question |  |  |
| --- | --- | --- | --- |
| **401** | Have you ever breast fed your current baby? | Yes  No | If yes go to question 402 |
| **402** | Has your infant fed breast milk in the last 24 hr? | Yes  No | If yes question no 403 if not question no 408 |
| **403** | If your infant breast fed in the last 24 hr, how many times did your infant breast fed? | ~~Times~~ mention frequency of breast feeding………….. |  |
| **404** | Has the infant received anything else other than breast milk, since breast feeding was initiated? | 1. Yes 2. No | If yes go to question 405 |
| **405** | what was other food/liquid that your infant fed in addition to breast milk | 1. ~~Plane~~ plain water 2. Fruit Juice 3. Fresh animal milk 4. Infant formula 5. Cereal gruel 6. Tea 7. Other specify______ |  |
| **406** | At what age did the child start complementary feeding? | ------------ month |  |
| **407** | Why did you give the baby these liquids/solids | 1, Advised by relatives/friends/neighbors  2, Advised by health care providers [ ]  3, Advised by TBA [ ]  4, Other (specify) |  |

| **408** | If your infant didn’t fed breast milk in the last 24 hr, what was the reason? | 1. Going back to work 2. I had illness 3. Infant illness 4. Decreased breast milk 5. I had no time 6. Because my infant was feeding infant formula 7. Other specify_____ |  |
| --- | --- | --- | --- |
| **409** | Did you put your infant at your chest immediately after you give birth? | 1. Yes 2. No | If yes go to question number 410 |
| **410** | How soon after birth did you put your infant for the first time to breast feed? | 1. Immediately after 1 hour of birth 2. After 1 hour up to 1 day of birth 3. After 3 days |  |
| **411** | If delayed more than 1 hr what was the reason that made you delayed in breast feeding initiation? | 1. C.s delivery 2. Maternal illness 3. Infant illness 4. Delayed breast milk secretion 5. Infant refusal 6. It is not appropriate time to initiate 7. Other specify _____ |  |
| **412** | What is the right time to give breast milk to a child after birth? | 1. Immediately 2. Within an hour 3. Between 1 hour and 3 hours 4. From 4 to 6 hours 5. From 7 to 24 hour 6. From 1 day to a week 7. More than a week 8. Never 9. I do not know |  |
| **413** | What is the right thing to do with the first milk or colostrum to anew born? | 1. Discard  2. Feed immediately |  |
| **414** | What is actually the right time to start complimentary foods in addition to the breast milk? | 1. 3 months or less  2. 4 months  3. 5 months  4. 6 months  5. 7 months or above |  |
| **415** | Which foods and or fluids are recommended to give a child under 6 months | 1. Only breast milk 2. Plain water 3. Infant formula (local example) and or milk, tinned, powder, or fresh animal milk   4. Juice or juice drinks  5. Yoghurt  6. Thinned  7. porridge  7. Any other fluids ____ |  |
| **416** | Giving breastfeeding immediately after birth is important | 1. strongly agree  2. agree  3. disagree  4. strongly disagree |  |
| **417** | Discarding the first milk or colostrum is not important before giving breast milk to the new born | 1. strongly agree  2. agree  3. disagree  4. strongly disagree |  |
